# Hematological and biochemical alterations in patients with suspected COVID-19 infection with negative and positive RT-PCR swabs

**DOI:** 10.3389/abp.2026.16764

**Published:** 2026-08-18

**Authors:** Lotfi S. Bin Dahman, Adnan A. Bakarman, Naela A. Al-Nakhbi, Ahmed M. Al-Haddad, Ammar H. Bin-Talib, Hamzah A. Al-Saadi, Suad T. Bashatah, Noora S. Al-Yazidi, Mohammed S. Al-Houti, Mohammed O. Bin-Dheyab

**Affiliations:** 1 Clinical Biochemistry Unit, Laboratory Medicine Department, College of Medicine and Health Sciences, Hadhramout University, Mukalla, Yemen; 2 Hadhramout Foundation-Human Development, Mukalla, Yemen; 3 Clinical Hematology Unit, Internal Medicine Department, College of Medicine and Health Sciences, Hadhramout University, Mukalla, Yemen; 4 Basic Medical Sciences Department, College of Medicine and Health Sciences, Hadhramout University, Mukalla, Yemen; 5 Medical Microbiology Unit, Medical Basic Sciences Department, College of Medicine and Health Sciences, Hadhramout University, Mukalla, Yemen

**Keywords:** biochemical parameters, COVID-19, hematological parameters, RT-PCR, Yemen

## Abstract

**Background:**

Hematological and biochemical abnormalities have been reported in patients with confirmed cases of coronavirus disease 2019 (COVID-19). This study compared selected hematological and biochemical parameters in patients with confirmed COVID-19 (RT-PCR positive) and suspected COVID-19 (RT-PCR negative).

**Patients and methods:**

A retrospective cross-sectional study was conducted among 106 patients admitted to the COVID-19 isolation centers at Ibn-Sina Hospital, Mukalla, Yemen, from 1 December 2021 to 30 October 2022. The study included 63 men and 43 women, with a mean age of 58.3 ± 17.5 years. Demographic, clinical, hematological, and biochemical data were extracted from medical records. The following were analyzed: complete blood count (CBC), random blood glucose (RBG), alanine transaminase (ALT), aspartate transaminase (AST), lactate dehydrogenase (LDH), gamma-glutamyl transferase (GGT), C-reactive protein (CRP), and ferritin.

**Results:**

Of the 106 patients, 58 (54.72%) had confirmed (RT-PCR positive) COVID-19, and 48 (45.3%) had suspected (RT-PCR negative) COVID-19. Compared with the RT-PCR-negative group, the RT-PCR-positive patients had significantly higher hemoglobin, hematocrit, red blood cell (RBC), and white blood cell (WBC) counts and were more likely to exhibit neutrophilia, lymphopenia, and eosinopenia. Serum LDH, GGT, CRP, and ferritin levels were also significantly higher in the RT-PCR-positive group. Using binary logistic regression analysis, LDH (OR: 1.008, 95% CI: 1.002–1.013, P = 0.004) and GGT (OR: 0.949, 95% CI: 0.903–0.997, P = 0.039) were independently associated with RT-PCR positivity.

**Conclusion:**

Among the investigated laboratory markers, LDH and GGT showed independent associations with RT-PCR positivity. Given the retrospective cross-sectional design, these findings should be interpreted as associations rather than evidence of predictive or causal relationships.

## Introduction

Coronavirus disease-2019 (COVID-19) is recognized as a pandemic caused by severe acute respiratory syndrome coronavirus 2 (SARS-CoV-2). Cases of this virus were first detected in Wuhan, China, in December 2019, and then spread rapidly to other countries. To date, it is responsible for infecting more than 767 million individuals and causing more than 7 million deaths (World Health Organization, 2023). According to WHO reports, 11,945 confirmed cases and 2,159 deaths were reported in Yemen from April 2020 to January 2023 (World Health Organization, 2026). The clinical characteristics of a COVID-19 infection range from asymptomatic in mild cases to acute respiratory distress syndrome and death in severe cases. The virus enters pulmonary epithelial cells via surface ACE2 receptors, causing pneumonia, followed by a systemic inflammation phase, then leading to respiratory failure and multi-organ dysfunction (Lippi and Henry, 2020). Common symptoms of a COVID-19 infection include headache, loss of smell, nasal congestion, cough, asthenia, myalgia, rhinorrhea, sore throat, fever, shortness of breath, nausea, and diarrhea (Qian et al., 2020; Xu et al., 2020; Wu et al., 2020). Additionally, hospitalized patients experienced persistent symptoms of fatigue, headache, dyspnea, and anosmia (Wu et al., 2020). Older age, increased body mass index (BMI), hypertension, diabetes, and cardiovascular disease have been reported as comorbidities of COVID-19 (Sudre et al., 2021; Bailly et al., 2022).

A COVID-19 infection is diagnosed using a reverse-transcriptase polymerase chain reaction (RT-PCR) assay by detecting the SARS-CoV-2 virus in saliva, nasal swabs, or throat swab specimens (Hafeez et al., 2020). However, different limitations of this assay have been reported. These include expensive equipment, a long turnaround time, and false-negative results (Xie et al., 2020; Li et al., 2020). Thus, there is a need for readily available tests to rapidly diagnose the disease.

Different routine hematological and biochemical parameters have been recorded by various studies. Additionally, patients with hematological abnormalities are at increased risk for various infections and other chronic diseases (Liu et al., 2020).

The severity of the infection can be predicted by leukocytosis, lymphocytopenia, neutrophilia, eosinopenia, thrombocytopenia, the neutrophil-to-lymphocyte ratio (NLR) (Fan et al., 2020; Alfadda et al., 2021; Chatterjee et al., 2024), and by elevated serum ferritin, C-reactive protein (CRP), alanine aminotransferase (ALT), aspartate aminotransferase (AST), and lactate dehydrogenase (LDH) (Alfadda et al., 2021). Other studies have also reported that hemoglobin and hematocrit levels are significantly higher among patients with positive RT-PCR results (Fan et al., 2020; Alfadda et al., 2021).

Serum ferritin and CRP levels were found to be significantly higher among positive RT-PCR patients compared with those with negative RT-PCR results (Alfadda et al., 2021). While the lungs are the primary target of COVID-19, this disease involves multiple other organs and cell types, including hepatocytes (Clark et al., 2021). One study reported that 11% of patients with COVID-19 had liver dysfunction, and 14%–53% showed elevated serum ALT and AST activities during the progression of the disease (Clark et al., 2021). Moreover, elevated ALT and AST levels were reported in a 2-month follow-up study of individuals discharged after COVID-19 infection (An et al., 2021). Thus, this study aimed to investigate these alterations in the hematological and biochemical parameters of patients suspected of having COVID-19 with positive and negative RT-PCR results.

## Materials and methods

### Study design

This is a retrospective cross-sectional study based on data from patients admitted to isolation centers and repeated negative RT-PCR tests to avoid false negatives, which ensures the reliability of the results (Elderdery et al., 2022), composed of 106 admitted patients in the isolation centers for COVID-19 of Ibn-Sina Hospital in Mukalla, Yemen, from 1 December 2021 to 30 October 2022.

### Patient selection

The study included all adult patients admitted to the COVID-19 isolation centers at Ibn Sina Hospital, Mukalla, Yemen, with either confirmed COVID-19 (RT-PCR positive) or suspected COVID-19 (RT-PCR negative) between 1 December 2021 and 30 October 2022. A total of 106 patients were enrolled, with 63 men and 43 women and a mean age of 58.3 ± 17.5 years. Based on SARS-CoV-2 RT-PCR results, patients were classified into two groups: 58 patients with confirmed COVID-19 (RT-PCR positive; 54.72%) and 48 patients with suspected COVID-19 (RT-PCR negative) (45.28%). Adult patients admitted with clinical suspicion of COVID-19 who underwent SARS-CoV-2 RT-PCR testing were eligible for inclusion. Patients with known liver disease, renal disease, hematological disorders, autoimmune diseases, or malignancies were excluded to minimize the influence of these conditions on the hematological and biochemical parameters investigated. Because of the retrospective nature of the study and the limited diagnostic resources available at our institution, systematic investigations for alternative respiratory pathogens or other inflammatory diseases were not routinely performed. Therefore, we cannot exclude the possibility that some RT-PCR-negative patients had other infectious or inflammatory conditions.

### Case definition

Based on the WHO’s SARS-CoV-2 case definition guidelines (World Health Organization, 2020), a suspected case of COVID-19 was defined as a patient with acute respiratory symptoms, including fever, cough, or shortness of breath. In the 14 days prior to symptom onset, the patient had to have met at least one of the following epidemiological criteria, with suspected cases subdivided into three components:

- component “A”: a recent history of travel to any of the affected countries;
- component “B”: a recent record of contact with confirmed or probable cases;
- component “C”: requiring hospitalization, without a clear diagnosis, regardless of any recent record of travel or contact with affected individuals. A confirmed case was defined as a suspected case with laboratory confirmation of COVID-19 infection by detecting SARS-CoV-2 through nasal/oropharyngeal swabs using RT-PCR assays. Patients with RT-PCR-negative results were assessed again within 48–72 h to avoid any false-negative results.

### Data collection

Data were extracted from the electronic medical files of each included patient using a predesigned questionnaire form. Socio-demographic characteristics, vital signs, comorbid conditions, clinical symptoms, laboratory parameters, treatment, and outcomes were retrieved. The study was approved by the Ethics Committee of the Medicine College, Hadhramout University, Yemen, according to the Declaration of Helsinki after the patients provided written informed consent (approval number: CM/REC5/6/2022).

### Blood sample collection and separation

Approximately 5 mm of venous blood was drawn from each participant. The blood sample was collected into two tubes. The first tube with ethylene diamine tetraacetic acid (EDTA) anticoagulant was used for complete blood count (CBC) measurement. The second tube, which contained no anticoagulant, was used to measure random blood glucose (RBG), ALT, AST, gamma-glutamyl transferase (GGT), LDH, CRP, and ferritin. The samples were left to clot for 30 min, after which the serum was separated and stored at −70 °C until analysis. To avoid repeated thawing and freezing, the serum was separated into aliquots. The CBC was determined using a hematology analyzer (Sysmex XS-500i, Bornbarch 1, 22848-Norderstedt, Germany). RBG, ALT, AST, GGT, LDH, and CRP were determined using a Cobas Integra 400 plus analyzer, and ferritin was determined using a Cobas e411 analyzer (Roche Diagnostics GmbH, Mannheim, Germany). Blood samples were collected by the medical laboratory technicians from the College of Medicine and Health Sciences, Hadhramout University. They were fully trained for this project.

### Statistical analysis

The Statistical Package for the Social Sciences (SPSS, version 25) was used for data analysis. The Shapiro–Wilk test was used to determine the normal distribution of the continuous variables. Categorical variables were presented as frequencies and percentages. Normal continuous variables were presented as the mean ± standard deviation (mean ± SD), while the median and interquartile range (IQR) were used for non-normal continuous variables. The differences between the studied groups were analyzed using an independent-samples t-test, a Mann–Whitney U test for non-normally distributed continuous variables, and a Chi-square test for categorical variables. Pearson correlation analysis was performed to test the correlation of the biochemical parameters. Odds ratios (ORs) and 95% confidence intervals (95% CIs) were used to evaluate the risk factors for developing COVID-19. Multivariable logistic regression analyses were conducted to identify independent risk factors for the disease. All statistical analyses were conducted at a 95% confidence level. A *P*-value <0.05 was considered to indicate statistical significance.

## Results

### Sociodemographic data and clinical characteristics of participants

Table 1 lists the sociodemographic data and clinical characteristics of the participants. Of the total participants (n = 106), 95.3% were over 45 years old, and the remaining (4.7%) were ≤45 years old, with a mean age of 58.3 ± 17.5 years. More than half of the participants (59.4%) were male, and 81.1% were married. Fever, cough, and sore throat were the most common symptoms among all patients, followed by breathing difficulty (99.1%), pneumonia (99.1%), joint and muscle aches (68.9%), and abdominal disorders (59.4%). Only 5.7% of the subjects had chills. Hypertension, diabetes mellitus, and lung disease were present in 94.3%, 80.2%, and 3.8% of the patients, respectively. No heart, liver, or renal diseases were reported. To compare the studied groups, 58 (54.72%) patients were RT-PCR-positive, and 48 (45.28%) were RT-PCR-negative. The mean age of the RT-PCR-positive patients was significantly higher than that of the RT-PCR-negative group (*P* = 0.001), and 53 (91.4%) of the RT-PCR-positive patients were over 45 years old (*P* = 0.013). There were significant differences between the two groups in terms of clinical symptoms of the disease, including joint and muscle aches (*P* < 0.001), abdominal disorders (*P* < 0.001), and chills (*P* = 0.006) in the RT-PCR-positive group compared to the RT-PCR-negative group. Additionally, the RT-PCR-positive patients had significantly higher percentages of diabetes mellitus and lung disease than the RT-PCR-negative group (*P* < 0.001 and *P* = 0.026, respectively). On the other hand, no significant differences between the two groups were observed in terms of breathing difficulty (*P* = 0.206), pneumonia (*P* = 0.206), or hypertension (*P* = 0.278).

**TABLE 1:** Sociodemographic data and clinical characteristics of participants.

Variable	Total (106)	RT-PCR-negative (48)	RT-PCR-positive (58)	*P*-value
Sociodemographic characteristics				
Age (years) mean ± SD	58.3 ± 17.5	52 ± 18.9	63.5 ± 14.5	0.001
Age group (years) ≤45 ≥46	5 (4.7%) 101 (95.3%)	20 (41.7%) 28 (58.3%)	5 (8.6%) 53 (91.4%)	0.013
Sex Male patients Female patients	63 (59.4%) 43 (40.6%)	28 (58.3%) 20 (41.7%)	35 (60.3%) 23 (39.7%)	0.834
Marital status Single Married	20 (18.9%) 86 (81.1%)	20 (41.7%) 28 (58.3%)	00 (00%) 58 (100%)	<0.001
Clinical symptoms				
Fever	106 (100%)	48 (100%)	58 (100%)	​
Cough	106 (100%)	48 (100%)	58 (100%)	​
Sore throat	106 (100%)	48 (100%)	58 (100%)	​
Difficulty breathing	105 (99.1%)	47 (97.9%)	58 (100%)	0.206
Joint and muscle aches	73 (68.9%)	17 (35.4%)	56 (96.6%)	<0.001
Nausea, vomiting and diarrhea	63 (59.4%)	6 (12.5%)	57 (98.3%)	<0.001
Chills	6 (5.7%)	00 (00%)	6 (10.3%)	0.006
Pneumonia	4 (3.8%)	0 (00%)	4 (6.9%)	0.206
Chronic diseases				
Hypertension	100 (94.3%)	44 (91.7%)	56 (96.6%)	0.278
Diabetes mellitus	85 (80.2%)	30 (62.5%)	55 (94.8%)	<0.001
Lung disease	4 (3.8%)	00 (00%)	4 (6.9%)	0.026

### Comparison of hematological parameters between RT-PCR-negative and RT-PCR-positive patients

Table 2 presents a comparison of biochemical parameters between patients with confirmed COVID-19 (RT-PCR positive) and patients with suspected COVID-19 (RT-PCR negative). Hemoglobin levels and Hematocrit values were significantly higher in RT-PCR-positive patients than in the RT-PCR-negative group (*P* = 0.004 and *P* = 0.018, respectively). Similarly, WBC and RBC counts were significantly higher among RT-PCR-positive patients (*P* < 0.001 and *P* = 0.044, respectively). Neutrophil counts were significantly increased in RT-PCR-positive patients (*P* < 0.001), whereas lymphocyte and eosinophil counts were significantly lower compared with the RT-PCR-negative group (both *P* < 0.001). In contrast, no statistically significant differences were observed between the two groups in mean corpuscular volume (MCV) (*P* = 0.950), mean corpuscular hemoglobin (MCH) (*P* = 0.153), mean corpuscular hemoglobin concentration (MCHC) (*P* = 0.079), platelet count (*P* = 0.395), monocyte count (*P* = 0.358), or basophil count (*P* = 0.322).

**TABLE 2:** Comparison of hematological parameters between RT-PCR-negative and RT-PCR-positive patients.

Parameter	RT-PCR-negative	RT-PCR-positive	*P*-value
Hemoglobin (g/dL)	11.5 ± 1.5	12.6 ± 2.1	0.004
WBC (x 10^3^/μL)	9.43 ± 4.73	17.56 ± 10.68	<0.001
RBC (x 10^6^/μL)	4.3 ± 0.7	4.6 ± 0.71	0.045
Hematocrit (%)	36.4 ± 5.1	38.9 ± 5.7	0.018
MCV (fL)	84.8 ± 7.3	84.9 ± 6.5	0.950
MCH (pg)	26.7 ± 2.8	27.5 ± 2.6	0.153
MCHC (g/dL)	31.4 ± 1.2	32.0 ± 2.1	0.079
Platelets (x 10^3^/μL)	258.7 ± 75.8	274.7 ± 109.9	0.395
Neutrophils (x 10^3^/μL)	70.01 ± 16.7	86.15 ± 7.1	<0.001
Lymphocytes (x 10^3^/μL)	21.16 ± 2.1	7.8 ± 4.6	<0.001
Monocytes (x 10^3^/μL)	7.23 ± 0.53	6.4 ± 0.74	0.358
Eosinophils (x 10^3^/μL)	1.2 ± 0.16	0.31 ± 0.06	<0.001
Basophils (x 10^3^/μL)	0.48 ± 0.06	0.37 ± 0.06	0.322

Data are expressed as mean ± SD. The mean difference in hematological parameters between patients with negative and positive RT-PCR results was analyzed using univariate analysis. A *P-value* of < 0.05 was considered significant. The analysis was performed at a 95% confidence interval.

### Comparison of biochemical parameters between RT-PCR-negative and RT-PCR-positive patients

Table 3 presents the comparison of biochemical parameters between patients with confirmed COVID-19 (RT-PCR positive) and patients with suspected COVID-19 (RT-PCR negative). Patients with positive RT-PCR results had significantly higher levels of random blood glucose (RBG), lactate dehydrogenase (LDH), gamma-glutamyl transferase (GGT), C-reactive protein (CRP), and ferritin than the RT-PCR-negative group (all *P* < 0.001). In contrast, no statistically significant differences were observed between the two groups in aspartate aminotransferase (AST) (*P* = 0.086) or alanine aminotransferase (ALT) (*P* = 0.077).

**TABLE 3:** Comparison of biochemical parameters between RT-PCR-negative and RT-PCR-positive patients.

Parameter	RT-PCT-negative	RT-PCR-positive	*P*-value
RBG (mg/dL)	81 (121–202)	67 (191–259)	<0.001
AST (U/L)	17 (21–39)	22 (24–46)	0.086
ALT (U/L)	19 (14–34)	18 (24–42)	0.077
LDH (U/L)	60 (163–223)	191 (320–512)	<0.001
GGT (U/L)	26 (26–52)	17 (72–89)	<0.001
CRP (mg/L)	3 (5.4–8.5)	50 (73–123)	<0.001
Ferritin (μg/L)	121 (269–390)	818 (872–1,691)	<0.001

Data are expressed as the median and interquartile range (IQR). The mean difference in biochemical parameters between patients with negative and positive RT-PCR results was analyzed using the Mann–Whitney U-test. A *P-value* of < 0.05 was considered significant. The analysis was performed at a 95% confidence interval.

### Pearson correlation analysis of CRP and ferritin levels in relation to liver enzyme levels among RT-PCR-positive COVID-19 patients

Table 4 presents the results of the Pearson correlation analysis between inflammatory markers (CRP and ferritin) and liver enzymes in patients with confirmed COVID-19 (RT-PCR-positive). CRP was positively correlated with AST (*r* = 0.208, *P* = 0.032), ALT (*r* = 0.281, *P* = 0.004), LDH (*r* = 0.540, *P* < 0.001), and GGT (*r* = 0.664, *P* = 0.045). Similarly, ferritin showed positive correlations with AST (*r* = 0.278, *P* = 0.004), ALT (*r* = 0.290, *P* < 0.001), LDH (*r* = 0.582, *P* = 0.001), and GGT (*r* = 0.623, *P* < 0.001).

**TABLE 4:** Pearson correlation analysis of CRP and ferritin levels in relation to liver enzyme levels among RT-PCR-positive COVID-19 patients.

​	CRP (mg/L)		Ferritin (ug/L)	
​	r	*P*-value	r	*P*-value
AST (U/L)	0.208*	0.032	0.278*	0.004
ALT (U/L)	0.281**	0.004	0.290**	<0.001
LDH (U/L)	0.540**	<0.001	0.582**	0.001
GGT (U/L)	0.664**	0.045	0.623**	<0.001

** Correlation is significant at the 0.01 level.

* Correlation is significant at the 0.05 level.

### Multivariable logistic regression analysis of CRP, LDH, and GGT independently associated with RT-PCR positivity

Table 5 presents the results of the multivariable logistic regression analysis evaluating the association between selected laboratory parameters and RT-PCR positivity. Among the variables included in the model, only LDH (OR: 1.004, 95% CI: 0.992–1.015, P = 0.004) and GGT (OR: 0.949, 95% CI: 0.903–0.997, P = 0.039) were independently associated with RT-PCR positivity.

**TABLE 5:** Multivariable logistic regression analysis of factors associated with RT-PCR positivity.

​	OR	(95% CI)	*P*-value
CRP (mg/L)	1.004	(0.992–1.015)	0.540
LDH (U/L)	1.008	(1.002–1.013)	0.004
GGT (U/L)	0.949	(0.903–0.997)	0.039

Data are presented as ORs (95% CsI) and analyzed by using multivariable logistic regression. Only the significant biochemical variables were included in the model analysis.

## Discussion

This study aimed to investigate hematological and biochemical parameters among patients with suspected COVID-19 according to SARS-CoV-2 RT-PCR status. Although both groups exhibited similar clinical presentations, patients with RT-PCR-positive results had a higher prevalence of COVID-19-related symptoms, including fever, sore throat, cough, pneumonia, and joint and muscle pain, compared with those who had negative RT-PCR results. In addition, lymphocyte counts were significantly lower among RT-PCR-positive patients, which is consistent with previous reports identifying lymphopenia as a common hematological feature associated with confirmed cases of COVID-19 (Terpos et al., 2020).

In the present study, lymphocyte counts were significantly lower among RT-PCR-positive patients than among RT-PCR-negative patients (*P* < 0.001). These findings align with previous studies demonstrating reduced lymphocyte counts in patients with confirmed COVID-19 (Terpos et al., 2020; Middleton et al., 2021; Karimi et al., 2021). Similarly, eosinophil counts were significantly lower among RT-PCR-positive patients (*P* < 0.001), whereas basophil counts showed no significant difference between the two groups (*P* = 0.322). Reduced eosinophil levels have been reported in patients with COVID-19 and may be associated with inflammatory responses and delayed recovery (Mao et al., 2021).

Conversely, WBC and RBC counts were significantly higher among RT-PCR-positive patients compared with RT-PCR-negative patients (*P* < 0.001 and *P* = 0.045, respectively). Neutrophil counts were also significantly increased in RT-PCR-positive patients (*P* < 0.001), consistent with previous studies that reported neutrophilia as part of the inflammatory response associated with SARS-CoV-2 infection (Alcántara-Alonso et al., 2021). Hemoglobin levels were significantly higher among RT-PCR-positive patients (*P* = 0.002), which is consistent with findings from a previous study conducted in Saudi Arabia (Alfadda et al., 2021). However, other studies have reported a progressive decline in hemoglobin levels with increasing disease severity among patients with COVID-19 (Fan et al., 2020). These differences may be related to variations in disease stage, severity, patient characteristics, and underlying comorbidities.

Alterations in liver-associated biochemical markers have frequently been reported among patients with COVID-19. In this study, LDH and GGT levels were significantly higher among RT-PCR-positive patients than among RT-PCR-negative patients (both *P* < 0.001), whereas AST and ALT levels did not differ significantly between the two groups (*P* = 0.086, *P* = 0.077, respectively). Elevated LDH levels may reflect tissue injury and systemic inflammation associated with SARS-CoV-2 infection, while increased GGT levels have been reported in relation to hepatobiliary involvement in patients with COVID-19 (Alfadda et al., 2021; Clark et al., 2021). However, previous studies have reported inconsistent findings regarding GGT alterations. Some investigations found no significant difference between confirmed and suspected cases, while others reported increased GGT levels among both RT-PCR-positive and RT-PCR-negative patients (Alfadda et al., 2021). These discrepancies may be explained by differences in study populations, comorbidities, disease severity, and diagnostic criteria.

Inflammatory biomarkers were also significantly elevated among RT-PCR-positive patients. Ferritin, an acute-phase reactant and marker of inflammation, was significantly higher in confirmed cases of COVID-19 (*P* < 0.001), consistent with previous studies reporting increased ferritin levels during SARS-CoV-2 infection (Gómez-Pastora et al., 2020). Elevated ferritin levels have been associated with immune activation and hyperinflammatory responses in patients with COVID-19. Similarly, CRP levels were significantly increased among RT-PCR-positive patients (*P* < 0.001), supporting previous evidence that CRP reflects systemic inflammation in patients with COVID-19 (Uzum and Turkkan, 2023).

In the multivariable logistic regression analysis, LDH and GGT were independently associated with RT-PCR positivity. However, these findings should be interpreted cautiously, as the model was based mainly on available laboratory parameters and did not include all potential clinical confounders. Therefore, LDH and GGT should not be considered independent predictors or causal risk factors for COVID-19 infection based on the current findings.

This study has several limitations. First, the retrospective cross-sectional design limits the ability to establish temporal relationships or causal inferences between the laboratory parameters and SARS-CoV-2 RT-PCR positivity. Second, the study was conducted at a single hospital with a relatively limited sample size; therefore, the findings should be interpreted with caution and may not be fully generalizable to other populations. Third, although multivariable logistic regression analysis was performed, the model was based mainly on available laboratory parameters and did not include all potential clinical confounders, such as age, comorbidities, and disease severity. Therefore, residual confounding cannot be completely excluded, and the observed associations between LDH, GGT, and RT-PCR positivity should not be interpreted as independent predictors or causal risk factors. Fourth, the RT-PCR-negative group included patients with clinical suspicion of COVID-19 infection but negative SARS-CoV-2 RT-PCR results. Due to the retrospective nature of the study and limited diagnostic resources, systematic screening for alternative respiratory pathogens or other inflammatory conditions was not routinely performed. Thus, the possibility that some RT-PCR-negative patients had other infectious or inflammatory diseases cannot be excluded. Finally, clinical outcomes such as disease severity, oxygen requirement, intensive care unit admission, mechanical ventilation, and mortality were not available for all patients and therefore could not be assessed. Further prospective multicenter studies with larger cohorts, comprehensive clinical characterization, appropriate adjustment for confounding factors, and evaluation of patient outcomes are needed to confirm these findings and determine their clinical relevance.

## Conclusion

This study identified significant differences in several hematological and biochemical parameters between patients with confirmed COVID-19 (RT-PCR positive) and patients with suspected COVID-19 (RT-PCR negative). Patients with confirmed cases of COVID-19 exhibited higher hemoglobin, hematocrit, RBC count, WBC count, neutrophil count, LDH, GGT, CRP, and ferritin levels, together with lower lymphocyte and eosinophil counts. In the multivariable logistic regression analysis, LDH and GGT were independently associated with RT-PCR positivity. However, given the retrospective cross-sectional design of the study, these findings should be interpreted as associations rather than evidence of predictive or causal relationships. Further prospective studies with larger sample sizes, appropriate adjustment for potential confounding factors, and comprehensive characterization of RT-PCR-negative patients are warranted to validate these findings and better understand the hematological and biochemical profiles of patients with suspected and confirmed COVID-19.

## Data Availability

The original contributions presented in the study are included in the article. Further inquiries can be directed to the corresponding author.
